# Hydroxypropyl Cellulose Polymers as Efficient Emulsion Stabilizers: The Effect of Molecular Weight and Overlap Concentration

**DOI:** 10.3390/gels11020113

**Published:** 2025-02-05

**Authors:** Diana Cholakova, Krastina Tsvetkova, Viara Yordanova, Kristina Rusanova, Nikolai Denkov, Slavka Tcholakova

**Affiliations:** Department of Chemical and Pharmaceutical Engineering, Faculty of Chemistry and Pharmacy, Sofia University, 1 James Bourchier Ave., 1164 Sofia, Bulgaria; dc@dce.uni-sofia.bg (D.C.); ktsvetkova80@gmail.com (K.T.); viordanova99@gmail.com (V.Y.); kneshkova@gmail.com (K.R.); nd@dce.uni-sofia.bg (N.D.)

**Keywords:** hydroxypropyl cellulose (HPC), emulsion, temperature stability, rheology, triglyceride oil, phase transition, coalescence

## Abstract

Hydroxypropyl cellulose (HPC) is a non-digestible water-soluble polysaccharide used in various food, cosmetic, and pharmaceutical applications. In the current study, the aqueous solutions of six HPC grades, with molecular mass ranging from 40 to 870 kDa, were characterized with respect to their precipitation temperatures, interfacial tensions (IFTs), rheological properties and emulsifying and stabilization ability in palm (PO) and sunflower (SFO) oil emulsions. The main conclusions from the obtained results are as follows: (1) Emulsion drop size follows a master curve as a function of HPC concentration for all studied polymers, indicating that polymer molecular mass and solution viscosity have a secondary effect, while the primary effect is the fraction of surface-active molecules, estimated to be around 1–2% for all polymers. (2) Stable emulsions were obtained only with HPC polymers with *M_w_* ≥ 400 kDa at concentrations approximately 3.5 times higher than the critical overlap concentration, c*. At PO concentrations beyond 40 wt. % or when the temperature was 25 °C, these emulsions appeared as highly viscous liquids or non-flowing gels. (3) HPC polymers with *M_w_* < 90 kDa were unable to form stable emulsions, as the surface-active molecules cannot provide steric stabilization even at *c* ≳ 4–5 c*, resulting in drop creaming and coalescence during storage.

## 1. Introduction

Cellulose is the most abundant natural polysaccharide. Although very hydrophilic, it is insoluble in water, due to the formation of intramolecular H-bonds. However, when one or several hydroxyl groups of the D-glycopyranose unit are etherified with substitutes like hydroxypropyl, hydroxypropyl methyl, ethyl methyl or others, hydrocolloids with different physicochemical properties are formed [1,2,3,4,5]. Some of these synthetically obtained cellulose derivatives are soluble in water and many of them have surface activity [1].

**Figure 1:**
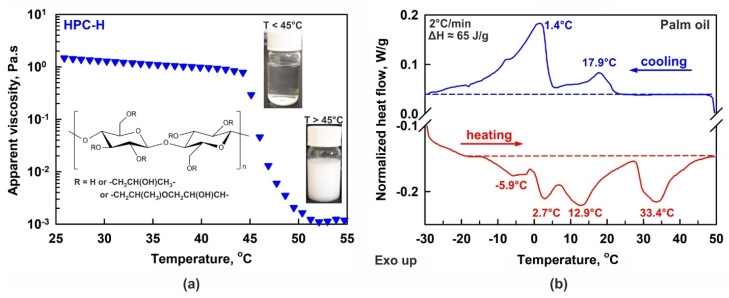
(**a**) Temperature dependence of 2 wt. % HPC-H aqueous solution viscosity. Below ca. 45 °C the solution is transparent and jelly-like, whereas the polymer precipitates and the viscosity decreases at higher temperatures. Inset: pictures of bottles filled with solution and general chemical structure of HPC. (**b**) DSC thermogram of bulk palm oil. The experiment was performed at 2 °C/min rate. The phase transition enthalpies measured upon both cooling and heating are in excellent agreement with each other, ΔH ≈ 65 J/g. The temperatures noted on the figure show the peaks maxima.

Hydroxypropyl cellulose (denoted hereafter as HPC) is a nonionic water- and ethanol-soluble polymer [1,6]. The HPC backbone consists of β-(1-4) linked D-glucopyranose units for which the hydroxyl groups at the C2, C3 and C6 atoms of the glucose ring are etherified with hydroxypropyl groups, -CH_2_CH(OH)CH_3_. The general structure of the HPC polymer is shown as an inset in Figure 1a. The molar substitution (MS) for HPC is defined as the number of hydroxypropyl groups attached per single glucose ring. As the hydroxypropyl group also contains one hydroxyl group, which may be etherified, the MS for HPC can be higher than 3 [3]. Such high molar substitution ensures very good solubility in water. In addition, the presence of hydrophilic and hydrophobic moieties in the HPC structure leads to its surface activity [1,7].

HPC is often used as a food additive (E463). The latest reports of the European Food Safety Authority (EFSA, see Refs. [8,9]) conclude that HPC with low and high molecular mass is non-digestible, does not raise any genotoxic concerns and has no carcinogenic properties or effect on reproductive performance, and it is therefore completely safe to use. Furthermore, results from daily exposure in the range of 6 g per day per person for a period of 8 months show that HPC is well tolerated and that there are no safety concerns associated with its intake [8].

HPC is used in beverages to improve the mouthfeel [1], in non-dairy whipped toppings and aerated desserts as a stabilizer [1], in Cellugel, which is applied as a book conservator [10], and in various other products as a thickener and gelling agent, stabilizer, film-forming agent or food additive [8,9,11,12]. It is also used for encapsulation and powder granulation [13,14,15]. HPC’s properties have been studied in relation to pharmaceutical, cosmetic and personal care applications [16,17,18,19,20]. Recently, the non-digestible properties of HPC and hydroxypropylmethyl cellulose (denoted hereafter as HPMC) have been studied in relation to the development of healthier foods with decreased lipid digestibility [21,22,23,24], such as panna cotta [25], cocoa cream [26] and muffins [27], and for their potential application in weight loss management.

Despite the numerous successful applications of HPC polymers, there are a limited number of studies related to their physicochemical properties and, more specifically, to HPC adsorption on oil–water interfaces, emulsifying ability and gelation properties. One of the main peculiarities, which should be considered when working with HPC aqueous solutions, is their relatively low precipitation temperature [28,29,30,31,32,33]. The precipitation temperature, *PT*, viz. the lower critical solution temperature or gelation temperature, is defined as the temperature below which the HPC molecules are hydrated by the water molecules, and at which the solution appears homogeneous and transparent; see the inset of Figure 1a. At temperatures above the *PT*, the formed H-bonds are no longer sufficient to hydrate the polymer chains well, and the polymer starts to precipitate, transforming the homogeneous aqueous solution into a suspension; see Figure 1a. The *PT* of the HPC polymers varies between 40 °C and 50 °C, depending on the HPC molar substitution and molecular mass [28,29,32].

The properties of HPC adsorbed on air–water and oil–water interfaces have been studied previously [7,34,35]. The authors showed that the equilibrium adsorption may be reached after several hours when low concentrations of polymer are used (*c* ≤ 10^−3^ wt. %), whereas the equilibrium was reached almost instantly at higher concentration of ca. 0.1 wt. %. The measured surface tensions at the air–water interface were about ≈41–45 mN/m, depending on the HPC grade studied. Furthermore, the HPC area per molecule in dense adsorption layers was found to be ca. 0.68 nm^2^ per monomer unit, whereas the area occupied by a monomer unit in the non-condensed adsorption layers was by ≈75% larger, ca. 1.19 nm^2^ [7]. Only about 64% of the monomer units were found to be positioned at the air–water interface, while the remaining units were located within the aqueous phase, next to the interface [7].

The emulsification properties of HPC also remain almost unexplored. Soybean oil emulsions with an oil volume fraction of 5%, stabilized by HPC and the anionic surfactant sodium dodecyl sulfate (SDS) were studied by Shimabayashi et al. [36]. They found that surface complexes were formed via hydrophobic interactions between the adsorbed HPC and SDS molecules. In another study, Mezdour et al. [37] explored 20 wt. % rapeseed emulsions stabilized by HPC alone or in combination with lecithin. These authors found that the drop size in the formed emulsions decreased significantly upon an increase in the polymer concentration from 0.1 to 1 g/L, despite the fact that the measured interfacial tension changed by 2 mN/m only. A steric stabilization of the drops was proposed, but the mechanisms explaining these results were not studied or discussed in detail. Ilyin et al. investigated concentrated pentadecane emulsions stabilized by HPC (*M_w_* = 140 kDa), cis-1,4-polyisoprene polymers and aggregates of nanoparticles [38]. The authors demonstrated that Pickering emulsions with visco-plastic behavior can be prepared by combining polymers and nanoparticles.

Several studies explored the emulsification properties of HPMC polymers [21,39,40,41,42]. The results from these studies could be used for comparison with the HPC-stabilized emulsions, as HPMC and HPC polymers have similar structures.

The major aim of the current study is to systematically investigate and compare the emulsifying properties of HPC polymers with molecular masses ranging from 40 to 870 kDa. The emulsions were characterized and their long-term stability was evaluated at two different temperatures (25 °C and 40 °C) in both gravitational and centrifugal fields. The main physicochemical factors controlling the emulsification process and emulsion stability are described and the destabilization mechanisms are discussed.

For emulsion preparation, palm oil (PO) was primarily used due to its widespread application in food and cosmetic products [43,44,45,46,47]. Additionally, its physicochemical properties present unique challenges in stabilizing PO-in-water emulsions with hydroxypropyl cellulose biopolymers, because the melting temperature of the palm oil (*T_m_* ≈ 40–45 °C) is very close to the *PT* of typical HPC polymers; see Figure 1. Consequently, a delicate balance between the polymer hydration and the liquid-to-solid fat content in the oil droplets is expected to significantly influence the emulsion’s properties and stability. This study was conducted at two temperatures, 25 °C and 40 °C. At 25 °C, about half of the oil froze, thus facilitating the partial drop coalescence and crystal network formation, whereas at 40 °C, the main fraction of the palm oil was in a liquid state. To evaluate the impact of oil freezing, sunflower oil was also tested for comparison, because it contains predominantly unsaturated triglycerides and has a significantly lower melting point (*T_m_* ≈ −17 °C) compared to PO.

## 2. Results and Discussion

### 2.1. Solution Characterization—PT and Rheological Properties

**Figure 2:**
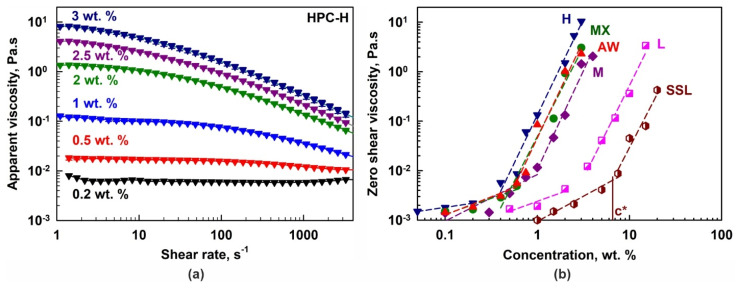
(**a**) Apparent shear viscosity as a function of the shear rate for HPC-H aqueous solutions with different polymer concentrations. (**b**) Zero shear viscosity as a function of HPC concentration for the different HPC grades studied. The symbol c* denotes the critical overlap concentration for the SSL polymer. The same approach was used for the determination of all c* concentrations.

The precipitation temperatures of the various HPC solutions were determined following the methodology described in Section 4.2.2. Illustrative results are presented in Figure 1a and Figure S1 in the Supplementary Materials. The measured precipitation temperatures, ranked from lowest to highest, are as follows (see Table 1): 42.6 ± 0.4 °C for HPC-AW, 44.3 ± 0.3 °C for HPC-H, 45.4 ± 0.7 °C for HPC-M, 48.3 ± 0.8 °C for HPC-MX, 49.6 ± 0.7 °C for HPC-L and 51.0 ± 0.8 °C for HPC-SSL. A trend of decreasing *PT* with increasing polymer molecular mass was observed, although slight deviations from the linear relation were noted for the MX and AW polymers. No significant dependence of *PT* on HPC concentration was detected within the concentration range explored in this study; see the concentration points presented in Figure 2b for the different polymers.

The dependence of the HPC-H solution viscosity on polymer concentration and shear rate is presented in Figure 2a. Viscosity increases significantly with polymer concentration, but decreases with increasing shear rate, indicating shear-thinning (non-Newtonian) behavior for concentrations *c* ≥ 0.5 wt. %. In contrast, at 0.2 wt. %, the HPC-H solutions behaved as a Newtonian fluid. Similar dependencies were observed for all other polymers; however, the concentration threshold for shear-thinning behavior depended on the polymer grade.

Using the measured rheological data and the Carreau–Yasuda model [48], the zero shear viscosities, *η_0_*, were calculated; see Figure 2b. The critical overlap concentrations, c*, were estimated from the crossover of the two linear regions in the log *η_0_* vs. log *c* plot [49]; see Table 1. As expected, c* increases with a decrease in the *M_w_* of the polymers: c* ≈ 0.4 wt. % for H, 0.6 wt. % for MX and AW, 0.9 wt. % for M, 2.9 wt. % for L and 6.5 wt. % for SSL.

To further characterize the viscoelastic properties of the solutions, we measured their storage (*G*′) and loss (*G*″) moduli; see Figure S2 in the Supplementary Materials. The results indicate that the loss moduli are consistently higher than the storage moduli across all systems studied, with a ratio of *G*″/*G*′ ≈ 3.8 ± 1.4. This result clearly demonstrates that the viscous behavior dominates over the elastic properties of the solutions at 40 °C.

These findings highlight significant differences in the properties of the studied HPC polymer grades. The observed variations suggest that the behavior of the emulsions stabilized with these polymer solutions could strongly depend on both the polymer concentration and the specific type of HPC used.

### 2.2. Characterization of Emulsion Properties

#### 2.2.1. Drop Size Distribution

**Figure 3:**
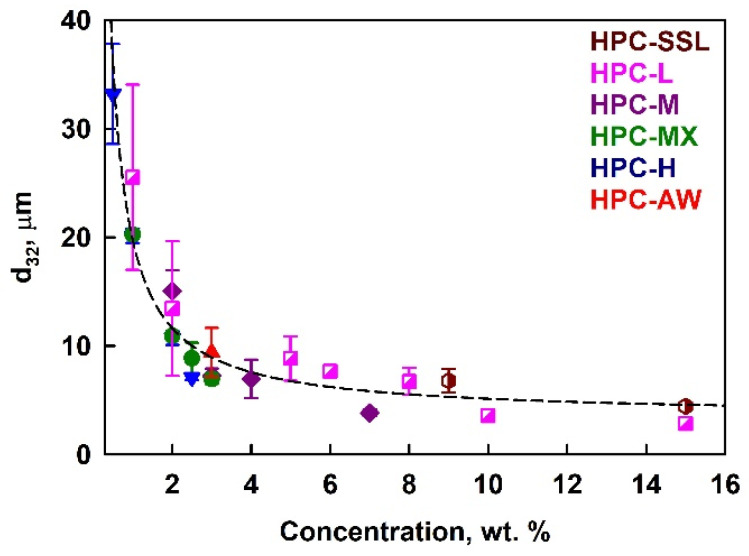
Average drop size in 25 wt. % palm oil emulsions as a function of the HPC polymer concentration in the aqueous phase at 40 °C temperature.

The results for the mean volume–surface diameter, *d*_32_, in PO emulsions stabilized by HPC polymers are presented in Figure 3. These emulsions were prepared with an oil weight fraction Φ_w_ = *m*_oil_/*m*_emuls_ = 0.25, corresponding to an oil volume fraction Φ = 0.27. The mean drop size decreased when increasing the polymer concentration, and no significant effect on the polymer type was observed. We note here that the polymer concentrations shown throughout the current paper are calculated with respect to the aqueous phase in the emulsion (not with respect to the whole emulsion).

This behavior can be quantitatively described using the model proposed by Tcholakova et al. [50]. Assuming that a threshold polymer concentration is required for steric stabilization of the emulsion droplets, and that the majority of the polymer is adsorbed on the droplet surfaces, the following expression can be used to predict the drop size in emulsions stabilized with different emulsifiers:

(1) $$d_{32}\approx \frac{6\Phi }{10\left(1-\Phi \right)}\frac{\Gamma }{c}.$$

Here, Φ is the oil volume fraction, Γ is the adsorption, expressed in mg/m^2^ and *c* is the emulsifier concentration in wt. %. Using this approach, we successfully described the experimental results and calculated the adsorption for all HPC polymers studied. The results are summarized in Figure S3a in the Supplementary Materials. When all data points were fitted together, the calculated average adsorption was remarkably high, approximately 100 mg/m^2^.

The estimated high polymer adsorption can be attributed to two possible effects: (1) the formation of a very thick polymer layer on the drop surface, or (2) a significant fraction of the polymer remaining in the aqueous phase without being adsorbed on the drop surface. To determine which of these two explanations applied to the systems studied, we performed rheological measurements on the emulsion serum, which was separated from the emulsions by 2–3 h of centrifugation at 40 °C. The visual appearance of the emulsions after centrifugation is shown in Figure S3b in the Supplementary Materials. The viscosities of the aqueous phase serum and with the HPC solution used for emulsion preparation were nearly identical; see Figure S3c in the Supplementary Materials. This result clearly indicates that the majority of the HPC molecules remain in the serum, with only a small fraction exhibiting surface activity and adsorbing onto the oil–water interface.

As previously reported [7], the typical area per monomer unit of HPC polymer at the air–water interface is about 0.68 nm^2^, with only around 64% of the monomer units actually being positioned at the interface after the polymers are spread on the solution surface. By comparing the calculated area per molecule from our experimental data with the 0.68 nm^2^ value in the literature, we estimated that only about 0.5% to 1.7% of the HPC molecules were adsorbed on the oil–water interface, while the main fraction of the HPC molecules remained dissolved in the aqueous phase. Most probably, the adsorbing components had an optimal ratio of hydrophobic to hydrophilic molecular fragments. Further dedicated study will be needed to clarify the actual adsorbing components in the various polymer systems, which is beyond the scope of the present study.

The latter result explains why no significant difference in viscosity was observed between the emulsion serum and the initial polymer solution. Note that in the present study, we have an oil–water interface, rather than an air–water interface as in [7].

To clarify whether the solution viscosity affects the drop size by changing the hydrodynamic conditions during emulsification, we plotted the drop diameters against solution viscosity; see Figure S4 in the Supplementary Materials. For emulsions prepared with a given polymer, an increase in viscosity resulted in smaller drops. However, no universal relationship was observed across the different polymers. At a viscosity of ≈60 mPa·s, the mean drop size for emulsions prepared with 15 wt. % HPC-SSL is 3 μm, whereas emulsion prepared with 1 wt. % HPC-H have a mean drop size of 20 μm; see Figure S4. These results demonstrate clearly that the primary factor determining the drop size distribution is not the viscosity of the aqueous phase, but rather the amount of the surface-active polymer species present in the aqueous solution. This polymer adsorbs onto the newly created oil–water interface, stabilizing the drops against coalescence. Notably, the master curve shown in Figure 3 is based on the total polymer concentration, not scaled by the critical overlap concentration, c*, implying that the surface-active fraction of the polymer is independent of its molecular mass. Above a certain polymer concentration, the mean drop size remains relatively constant, as there is a sufficient amount of surface-active polymer to cover the entire surface of the drops created under the given hydrodynamic conditions. Note that the decrease in the drop size at higher HPC concentrations with the increase in solution viscosity is relatively small. The 5.6-fold increase in solution viscosity (from 0.06 Pa·s to 0.34 Pa·s) leads to a 1.3-fold decrease in *d*_32_ (from 4.4 μm to 3.4 μm). This result evidences that the emulsification occurs in the transition zone between the two limiting regimes of turbulent emulsification—the turbulent inertial regime (drop size does not depend on solution viscosity) and turbulent viscous regime (drop size decreases with the inverse square root of solution viscosity); see Refs. [50,51,52,53] for explanations of these regimes.

#### 2.2.2. Viscosity of the Emulsions

**Figure 4:**
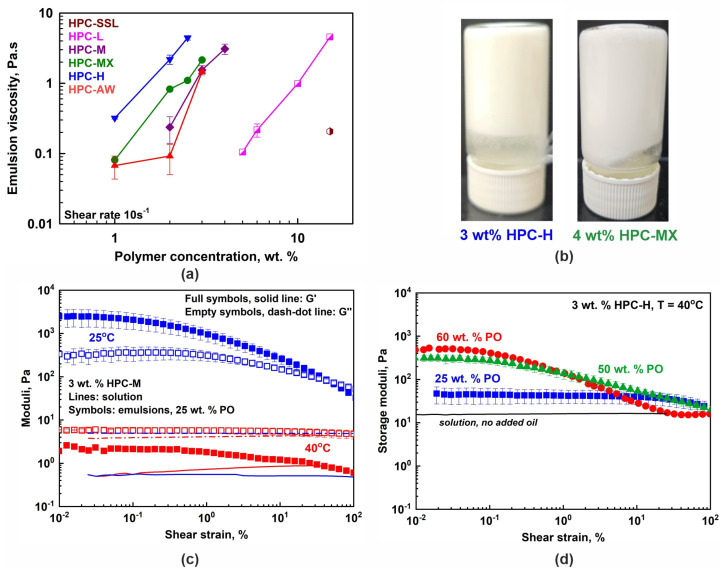
(**a**) The 25 wt. % PO emulsion viscosities measured at a 10 s^−1^ shear rate as a function of HPC concentration. The viscosity increases with the increase in polymer concentration, mainly due to the increase in the viscosity of the respective aqueous solution. Data obtained at 40 °C. (**b**) Illustrative pictures showing the non-flowing gel-like emulsions prepared with 3 wt. % HPC-H or 4 wt. % HPC-MX, for 25 wt. % PO-in-water emulsions stored at 25 °C. (**c**) Storage (*G*′, full symbols) and loss (*G*″, empty symbols) moduli measured for 3 wt. % HPC-M emulsion with 25 wt. % PO at 25 °C (blue symbols) and 40 °C (red symbols). The moduli increase their values upon cooling due to the partial PO freezing. The lines show the respective HPC-M solution moduli (*G*′, solid line and *G*″, dash–dot line). (**d**) Storage moduli dependence on PO concentration. All samples are prepared with 3 wt. % HPC-H polymer and the rheological properties are measured at 40 °C. The solid black line represents the storage modulus of the 3 wt. % HPC-H solution.

The rheological properties of the emulsions containing 25 wt. % PO were also studied; see Figure 4a and Figure S5a. Shear-thinning behavior was observed for all emulsion samples studied at 40 °C. Furthermore, the emulsion viscosity measured at 40 °C was no more than two to three times higher than the solution viscosity when determined at a shear rate of 10 s^−1^ (see Figure S5b), indicating that there were no significant interactions between the droplets in these emulsions. The viscosity ratio decreased to 1.4 ± 0.7 at $\dot{\gamma }$ = 100 s^−1^ at 40 °C. Therefore, we conclude that the rheological properties of the emulsions at 40 °C are predominantly governed by the rheological characteristics of the respective aqueous solutions used in emulsion preparation. Note that the oil volume fraction in most of the studied emulsions is much lower (25 wt. % ≈ 27.2 vol. %) compared to the volume fraction of closely packed drops, which is ≈64 vol. % for non-deformed monodisperse spheres and ≈72 vol. % for polydisperse drops [54,55].

However, the properties changed significantly when studied at 25 °C due to the partial freezing of the palm oil droplets. Illustrative results are presented in Figure 4b,c. The stable emulsion samples observed at 25 °C appear either as highly viscous liquids or as non-flowing gels even when prepared with 25 wt. % PO. Their viscosities and storage and loss moduli significantly increased, compared to those observed at 40 °C. Furthermore, a similar increase in the viscoelastic properties of the prepared emulsion samples was observed when higher concentrations of oil were used (50 or 60 wt. %), as illustrated in Figure 4d for samples studied at 40 °C. These results demonstrate that both temperature decrease and oil weight fraction increase can be used to finely tune the rheological properties of the prepared emulsions.

### 2.3. Emulsion Stability

The emulsions studied in this work can be used as prototypes for the formulation of various food and cosmetic products. A key requirement for these applications is their long-term stability, ideally lasting for several weeks, and preferably extending up to 1 or even 2 years. In this section, we present our findings on the long-term stability of the prepared emulsions, examining how they are affected by storage conditions. Additionally, we compare the results from stability tests conducted under gravitational conditions with those obtained upon centrifugation.

#### 2.3.1. Stability in Gravity Field

The stability of the emulsions during storage was studied at two temperatures: 25 °C and 40 °C. The temperature of 25 °C represents a typical storage condition for the product; however, a significant fraction of the PO is in a frozen state, as demonstrated by the DSC thermogram in Figure 1b and by the microscopy images in Section 2.4.3. To assess the effect of partial drop freezing at 25 °C, experiments were also conducted at 40 °C, where the main part of the PO was in a liquid state. Higher temperatures were not tested due to the precipitation of the HPC polymers at 43–51 °C, as discussed in Section 2.1 above.

**Figure 5:**
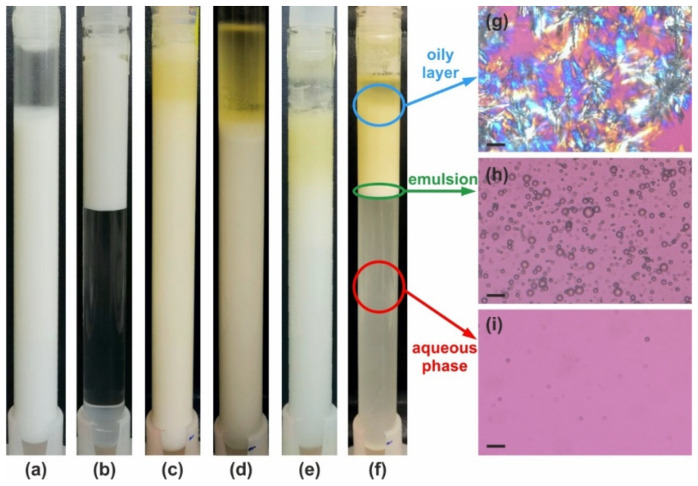
Illustrative examples of emulsion appearance after 28 days of storage at 40 °C. (**a**–**f**) Examples for the six main types of behavior as defined in Table 2. The emulsions are prepared with 25 wt. % PO and stabilized by (**a**) 2.5 wt. % HPC-MX—stable emulsion; (**b**) 3 wt. % SDS—stable with respect to coalescence, but intensive creaming is observed; (**c**) 2 wt. % HPC-M—partial coalescence in the upper layer; (**d**) 20 wt. % HPC-SSL—bulk oil layer is formed on the top; (**e**) 1 wt. % HPC-H—creaming and partial coalescence; and (**f**) 2 wt. % HPC-L—creaming and coalescence. (**g**–**i**) Optical microscopy images obtained at 25 °C in polarized light, showing the content of different layers in the sample shown in (**f**): (**g**) oily layer—crystallized PO is observed; (**h**) emulsion layer; and (**i**) aqueous phase layer in which only the smallest oily drops have remained, making it slightly turbid. Scale bars = 20 μm.

Stability tests were performed with palm oil emulsions stabilized by the various HPC grades. To summarize the obtained results, we categorized the samples on the basis of several distinct destabilization mechanisms which were observed: (1) drop creaming; (2) partial drop coalescence, identified by the appearance of a yellowish layer on top; and (3) complete drop coalescence, indicated by the formation of a bulk oil layer on top of the emulsion. The most stable samples exhibited none of these destabilization mechanisms. Illustrative pictures showing the appearances of samples across these categories are shown in Figure 5, while Table 2 summarizes the results for the different HPC grades studied. Further illustrative results and details about the performed stability evaluation are presented in Appendix A.

As expected, the most stable emulsions were those with the smallest droplet sizes and the highest viscosity of the aqueous phase. The minimum polymer concentrations required to prepare completely stable emulsions, stored for 1 month at 40 °C, were 3 wt. % for HPC-M, 2 wt. % for HPC-H and 2 wt. % for HPC-MX. In contrast, partial coalescence, complete coalescence and/or creaming were observed in all emulsions stabilized by HPC-SSL at concentrations up to 20 wt. %, HPC-L at concentrations up to 15 wt. % and HPC-AW at concentrations up to 3 wt. %.

It should be noted that although the concentration difference studied, Δ*c* = *c* − c*, was substantially higher for the lower-molecular-weight emulsifiers (≈13 wt. %), it did not ensure the formation of stable emulsions. Conversely, stable emulsions were prepared at Δ*c* ≈ 1.5–2 wt. % for the emulsifiers with higher molecular weight, except for HPC-AW.

The stability of the best emulsions was also monitored over an extended period. For the 2 wt. % HPC-H emulsion, minimal creaming and coalescence were observed after 6 months of storage. Emulsions stabilized with 3 and 4 wt. % HPC-M remained completely stable for 3 months, but the 3 wt. % emulsion exhibited a minimal separation of oil (less than 0.1 mL, corresponding to ca. < 2% of the oil in the sample) after 5 months. In emulsions stabilized by HPC-MX emulsion, partial creaming was observed after 3 months at 2 to 3 wt. % concentration, and a small amount of separated oil appeared on the sample surface after 6 months of storage at 40 °C.

To compare the emulsifying properties of HPC polymers with those of the commonly applied low-molecular-mass surfactants and other standard polymers, additional experiments were conducted with 1 to 3 wt. % SDS, 3 to 5 wt. % Tween 80^®^, 6 wt. % carboxymethyl cellulose (CMC), 3 wt. % HPMC-A and 1 wt. % HPMC-B as emulsifiers for 25 wt. % PO-in-water emulsions. Although the aqueous phases prepared with HPMC-B had viscosities similar to those of 3 wt. % HPC-M and 2 wt. % HPC-H at $\dot{\gamma }$ = 1 s^−1^, the HPMC-B solutions exhibited significantly more pronounced shear-thinning behavior. At a shear rate of 10 s^−1^, their viscosity was 4–5 times lower than that of HPC-H and HPC-M, respectively; see the comparison in Figure S6a in the Supplementary Materials. The PO emulsions prepared with 1 wt. % HPMC-B were unstable during storage, exhibiting both partial creaming and coalescence. Similar instability was observed with 3 wt. % HPMC-A emulsions. The viscosity of the 6 wt. % CMC solution was comparable to that of 2 wt. % HPC-H. Respectively, emulsions stabilized with CMC demonstrated relatively good stability during storage, showing only minimal creaming and coalescence, even after 4 months of storage at 40 °C.

The concentrations of low-molecular-mass surfactants were carefully selected to ensure that the emulsification occurred in the plateau region of the *d*_32_ vs. *c* dependence; see Figure 3 and Ref. [50]. The mean volume–surface diameter for the PO emulsion stabilized by 3 wt. % SDS was 11.6 ± 0.7 μm, while emulsions stabilized by 3 wt. % and 5 wt. % Tween 80^®^ showed similar droplet sizes, *d*_32_ ≈ 18.9 ± 0.5 μm. The SDS-stabilized emulsions were stable against drop–drop coalescence but were prone to creaming; see Figure 5b. In contrast, Tween 80^®^ surfactant was unable to stabilize the prepared emulsions against both creaming and partial coalescence. Destabilization of Tween 80^®^-stabilized samples occurred rapidly, and partial gravitational separation and the onset of partial coalescence were evident after just one day of storage.

These results highlight the potential of the high-molecular-mass HPC polymers for the preparation and stabilization of triglyceride oil emulsions, even when the drops contain both solid and liquid oil fractions. Emulsions stabilized with HPC remained entirely stable at 40 °C for at least one month when prepared with HPC-M at *C* ≥ 3 wt. %, HPC-MX at *C* ≥ 2 wt. % and HPC-H at *C* ≥ 2 wt. %; see Table 2 and Figure 5a. Furthermore, at lower temperatures these samples were non-flowing gels, allowing fine-tuning of the desired rheological behavior, depending on the specific emulsion application.

#### 2.3.2. Stability upon Centrifugation

The stability of the best emulsions, identified in the gravity field experiments, was further investigated using centrifugation. Previous studies have suggested centrifugation as a very efficient accelerated stability test [56,57,58,59].

**Figure 6:**
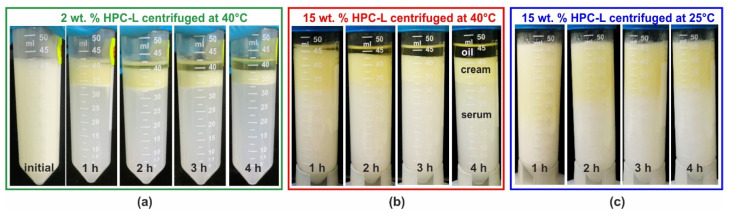
Results from centrifugation experiments performed with 25 wt. % PO emulsions, stabilized by HPC-L polymer. (**a**) The 2 wt. % HPC-L, 40 °C centrifugation temperature, with emulsion appearance shown before centrifugation and after 1, 2, 3 and 4 h centrifugation; (**b**) the 15 wt. % HPC-L at 40 °C; and (**c**) the 15 wt. % HPC-L at 25 °C.

Figure 6 presents illustrative images of the emulsion appearance before centrifugation and after 1, 2, 3 and 4 h of centrifugation. These results were obtained with 25 wt. % PO emulsions stabilized by HPC-L polymer. As shown in Figure 6a, at a polymer concentration of 2 wt. %, complete creaming and partial coalescence are evident even after the first hour of centrifugation. After an additional 3 h, the emulsion is almost completely destroyed with approximately half of the oil separated as a bulk phase above the emulsion. For comparison, the emulsion appearance after 1 month of storage under steady conditions closely resembles that observed after 2 h of centrifugation.

The formation of a bulk oil layer, resulting from drop–drop coalescence during centrifugation, is also observed at the highest HPC-L concentration studied, 15 wt. %. For this sample, even after 4 h of centrifugation at 40 °C, the lower phase remains turbid, indicating the presence of numerous emulsion droplets, scattering light in the aqueous serum. However, the amount of separated bulk oil after 4 h becomes comparable to that observed at the lower HPC-L concentration, cf. Figure 6a,b. When the same experiment is performed at 25 °C, as shown in Figure 6c, similarly to the experiments conducted under a gravitational field, a yellowish layer of partially coalesced drops appears on top of the emulsion, while no bulk oily layer is observed to separate. However, upon heating, the coalescence is enabled and proceeds. A comparison of the centrifugation results for several additional samples at 25 °C is presented in Figure S7 in the Supplementary Materials.

**Figure 7:**
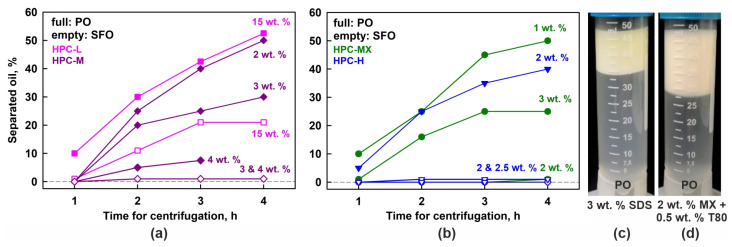
Results obtained in centrifugation experiments at 40 °C. (**a**,**b**) Percentage of separated oil, as a function of centrifugation time. The results for palm oil emulsions are given with full symbols and the results for sunflower oil with empty symbols. (**c**,**d**) Emulsions with 25 wt. % PO stabilized by 3 wt. % SDS (**c**) or 2 wt. % HPC-MX + 0.5 wt. % T80 (**d**). After 4 h of centrifugation, these samples experienced only creaming, while no drop coalescence or formation of bulk oily layer was observed.

Systematic centrifugation experiments at 40 °C were also performed using HPC-M, MX and H polymers as emulsifiers. These results are summarized in Figure 7a,b. The observed trends closely resemble those in the gravity experiments—emulsions with higher polymer concentrations exhibit better stability. However, even at the highest HPC-M concentration of 4 wt. %, a very thin oily layer of coalesced drops formed during centrifugation, whereas the same sample remained completely stable for over 3 months under a gravity field.

Additionally, the centrifugation stability of PO emulsions stabilized by SDS was studied. Similarly to the observations during storage under gravity, this sample exhibited only creaming, with no evidence of drop coalescence; see Figure 7c.

To evaluate whether combining a small amount of nonionic surfactant with the HPC polymer could enhance the emulsion stability, we performed additional experiments using 25 wt. % PO dispersed in 2 wt. % aqueous solution of HPC-MX and 0.5 wt. % Tween^®^ surfactant (both Tween 20^®^ and Tween 80^®^ were tested). Similar to the case with SDS, these emulsions exhibited only drop creaming upon centrifugation, without any signs of drop coalescence, as shown in Figure 7d. Comparable results were obtained under gravitational conditions—all tested Tween grades (20, 40, 60 and 80) yielded stable emulsions with respect to drop coalescence. Notably, these emulsions remained stable for at least one month with respect to coalescence, although the drops quickly creamed to the top of the emulsions.

We also investigated whether adding Tween surfactants into the HPC-MX solution significantly reduced the viscosity of the aqueous phase. Indeed, the viscosity of the 2 wt. % HPC-MX solutions decreased by 2.5 to 3.5 times upon the addition of 0.5 wt. % Tween surfactant; see Figure S6b in the Supplementary Materials. This viscosity reduction was most probably caused due to H-bonding between HPC and Tween molecules, as previously suggested [35], which disrupts the HPC network and decreases the solution viscosity. The mechanism of emulsion stabilization also differs in the presence and absence of Tween surfactants, as discussed further in Section 2.4.2 below.

Finally, to study the effect of partial freezing of the oily phase on the drop coalescence, we prepared and studied emulsions with sunflower oil (SFO) as the dispersed phase. Sunflower oil triglycerides contain approximately 90% unsaturated chains and only about 10% saturated chains, resulting in a much lower melting temperature compared to palm oil, *T_m_* ≈ −17 °C for SFO versus ≈40 °C for complete melting of PO. Therefore, the SFO drops remained in the liquid state under all studied conditions.

The results from these experiments are presented with empty symbols on the graphs shown in Figure 7a,b. It is evident that no coalescence occurs in the SFO emulsions, stabilized by 2 wt. % HPC-H, 2 or 3 wt. % HPC-MX, and 3 or 4 wt. % HPC-M. In contrast, the 15 wt. % HPC-L SFO emulsion forms a bulk oily layer, although it is much thinner than the layer observed in the corresponding PO samples. After 4 h of centrifugation, approximately 50% of the oil in the PO emulsions separated into a bulk layer, whereas only about 20% of the oil separated in the SFO emulsions. This comparison suggests that the partially frozen drops enhance the destabilization process significantly.

The main conclusions from these experiments are as follows: (1) Stable emulsions are formed from HPC polymers with *M_w_* ≳ 400 kDa at concentrations around 3.5 × c*. The only exception is HPC-AW, which is unable to stabilize the emulsions. (2) Polymers with *M_w_* < 80 kDa cannot stabilize the emulsions, even at concentrations around 4–5 × c*.

### 2.4. Mechanism of Emulsion Destabilization

To explain the observed trends, we performed a series of model experiments, aiming to identify the key differences between the various HPC polymer grades. These experiments involved measuring the interfacial tension at the aqueous solution–oil interface, performing optical observations of thin foam and emulsion films in a capillary cell, and carrying out model optical observations of emulsion samples during cooling and heating. The results from these experiments are presented and discussed in the current section.

#### 2.4.1. Interfacial Tensions (IFTs)

**Figure 8:**
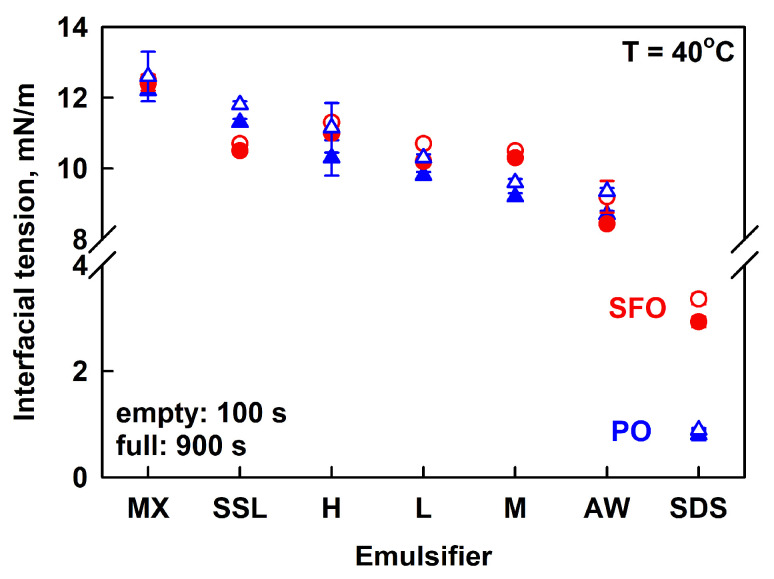
Interfacial tensions measured after 100 s (empty symbols) and 900 s (full symbols) for palm oil (blue triangles) or sunflower oil (red circles) pendant drops, immersed in 1 wt. % HPC solution (SSL, L, M, MX, H, AW) or 3 wt. % SDS. The emulsifiers are arranged in the order of descending IFT.

The interfacial tension of the various polymers was measured using a pendant drop of PO or SFO, immersed in 1 wt. % HPC aqueous solutions. All measurements were performed at 40° C to ensure that PO was melted. The measured IFT values were similar for all HPC solutions, ranging from ca. 12.5 mN/m for HPC-MX to 9 mN/m for HPC-AW; see Figure 8 and Figure S8 in the Supplementary Materials. The IFT decreased in the following order: MX > SSL > H > L > M > AW. It is seen that there is no direct correlation between the emulsion stability and the IFT values. Emulsions with HPC-AW and SSL were unstable, while emulsions with HPC-M and HPC-MX remained stable for several months.

The main conclusion from these experiments is that all polymers contain surface-active species capable of adsorbing at the PO–water and SFO–water interfaces, thereby reducing the interfacial tension. SDS, however, was found to reduce IFT to a greater extent than all studied polymers, probably due to the formation of a denser adsorption layer at the oil–water interface. As a result, SDS can stabilize the emulsion drops against coalescence. However, the lower viscosity of the aqueous phase causes emulsion drops to cream over time in the SDS-stabilized emulsions.

#### 2.4.2. Film Stability

**Figure 9:**
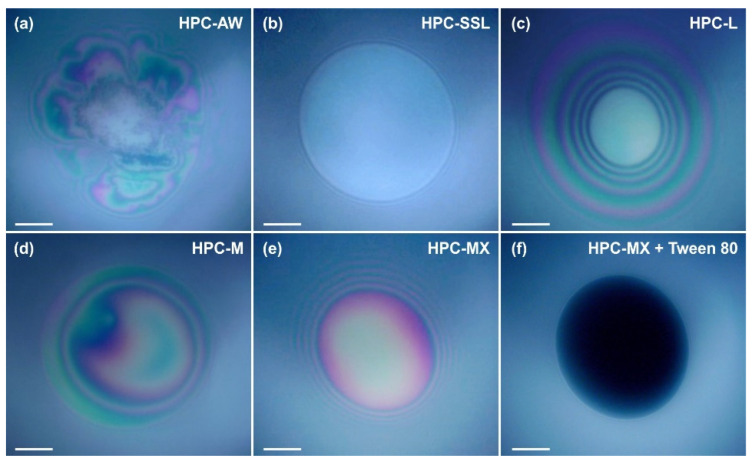
Foam films observed in reflected light in the Scheludko cell. The HPC concentrations are 1 wt. % for AW, M and MX (**a**,**d**,**e**), 2 wt. % for SSL and L (**b**,**c**) and 1 wt. % for MX + 0.25 wt. % Tween 80^®^ mixture in (**f**). The films shown in (**a**–**c**) are unstable and broke, whereas those shown in (**d**–**f**) remained stable for more than 15 min. Time elapsed between the film formation and taking the pictures: (**a**) 10 min, (**b**) 2 min, (**c**) 10 min, (**d**) 10 min, (**e**) 10 min, (**f**) 10 min. Scale bars = 50 μm.

When two emulsion drops come into close contact with each other, a liquid film forms between them. The stability of this film determines the stability of the drops against coalescence. To investigate the behavior of liquid films formed from polymer solutions, we performed model experiments using a Scheludko capillary cell [60,61,62]; see also Section 4.2.8 for a brief description of this experiment. We observed both foam films (formed between two air–water interfaces) and emulsion films (formed between two oil–water interfaces). Illustrative images from these experiments are presented in Figure 9.

The experiments show that a 2 wt. % SSL concentration is insufficient to stabilize the foam and emulsion films. Foam films thin down to a thickness of *h* ≈ 70–80 nm and rupture, whereas the emulsion films rupture during the thinning process at *h* > 100 nm. This behavior shows that the surface-active species in the 2 wt. % SSL can adsorb on the oil–water interface and reduce the interfacial tension. However, they are unable to provide a sufficient steric stabilization of the films formed between the emulsion drops. As a result, the respective emulsions are highly unstable and a bulk oil layer separates on the emulsion surface during storage.

Experiments with 2 wt. % HPC-L show that these films are also unstable, but the required time for destabilization is ≈4 min, compared to 0.5 min for films stabilized by HPC-SSL. Note that the viscosity of 2 wt. % HPC-L is only twice that of 2 wt. % HPC-SSL, suggesting that the eightfold increase in the lifetime, observed with HPC-L-stabilized films, cannot be explained by the increased viscosity only. Instead, it is related to a long-range steric repulsion, acting between the film surfaces in films formed from 2 wt. % HPC-L solutions. This repulsion slows down the rate of film thinning and increases the film stability. The better stabilization of HPC-L films compared to HPC-SSL films is in good agreement with the higher stability of emulsions formed from HPC-L, compared to those stabilized by HPC-SSL.

The experiments with 1 wt. % HPC-M show that this polymer is inefficient at stabilizing the foam films, despite the significantly higher viscosity of 11 mPa·s of the respective solution. Around 70% of the foam films rupture around 5 min after their formation at *h* ≈ 70 nm, while 30% of the films remain stable for over 10 min. The stable films typically have a thickness greater than 100 nm. The fact that a fraction of the foam films rupture whereas the others remain stable suggests that the concentration of surface-active species capable of inducing steric repulsions between the film surfaces is relatively low, leading to film rupture. The emulsion films remained stable for more than 10 min and their thickness was *h* > 100 nm. The better stabilization of these emulsion films is associated with their slower thinning, driven by the lower interfacial tension of the oil–water interface (as compared to the surface tension of the air–water interface).

The experiments performed with 1 wt. % HPC-MX show that all films (both foam and emulsion) remain stable for over 10 min with *h* > 100 nm. This stability is attributed to the higher viscosity of the polymer solution, approximately 90 mPa·s, and the long-range steric repulsion, induced by the adsorption of surface-active molecules on the film surfaces.

In contrast, the experiments with 1 wt. % HPC-AW, which has a viscosity similar to HPC-MX, reveal that the viscosity alone is insufficient to stabilize the films. The foam films formed with HPC-AW solution contain numerous entrapped aggregates. These aggregates stabilized the foam films and they had a lifetime > 10 min. However, when emulsion films were formed, they were highly unstable and ruptured within less than 5 min. This comparison indicates that the observed surface-active aggregates stabilized the foam films, without having such an effect on the emulsion films.

Additional experiments with 1 wt. % HPC-MX combined with 0.25 wt. % Tween 80^®^ showed that the behavior of the respective films was controlled by the surface-active molecules adsorbed on the film surfaces. The presence of Tween 80^®^ leads to a slight reduction in the solution viscosity, but it reduces the film thickness significantly down to ≈20 nm. This result suggests that Tween 80^®^ replaces the surface-active molecules from HPC-MX in the adsorption layer. The resulting films are highly stable, correlating with the good stability of the respective emulsions against coalescence. However, due to the formation of thinner films, the droplets tend to aggregate and cream to the top of the emulsion.

#### 2.4.3. Optical Observations

**Figure A1:**
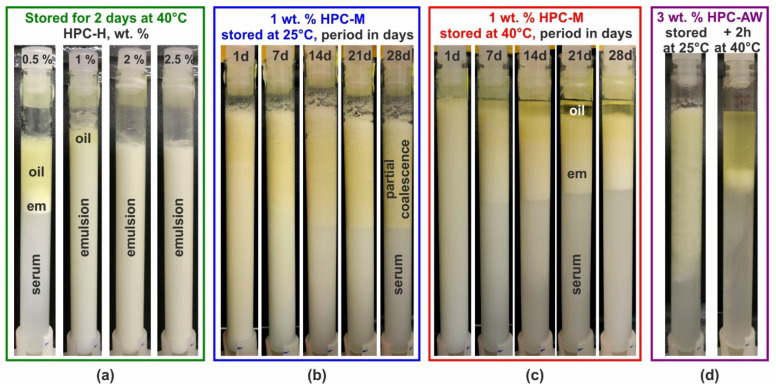
Visual appearance upon storage of emulsions prepared with 25 wt. % PO stabilized by HPC polymers. (**a**) Emulsions stabilized by HPC-H solutions with different concentrations as noted on the images. The samples were stored for 2 days at 40 °C. Partial coalescence and creaming is observed in the sample with 0.5 wt. % polymer, and a thin layer of partially coalesced drops is formed on top of the 1 wt. % emulsion, whereas the 2 and 2.5 wt. % emulsions are stable and no changes are seen compared to the initial emulsions. (**b**,**c**) The 1 wt. % HPC-M emulsions stored at 25 °C (**b**) and at 40 °C (**c**) for a period of 1 to 28 days. (**d**) The 3 wt. % HPC-AW emulsion stored for 71 days at 25 °C (left picture). Although this emulsion does not seem completely destroyed, after melting the PO for 2 h at 40 °C, the main fraction of the oil phase separates as a bulk layer on top of the container (right-hand-side image).

To directly illustrate the process of drop–drop coalescence in PO emulsions, we performed two different types of experiment. First, we visualized the processes occurring in a sample pre-stored at 25 °C during its heating to 40 °C, i.e., we microscopically visualized the macroscopic process shown in Figure A1 in Appendix A. For this experiment, we took a sample from the cream of an emulsion stored at 25 °C and placed it as a thin layer between two cover glass slides (100 μm thickness). The sample was then heated at a rate of ≈1.75 °C/min until complete oil melting occurred, while being observed under an optical microscope. The results from this experiment are presented in Figure S9 in the Supplementary Materials and Supplementary Video S1.

At 25 °C, the background of the sample had bright colors due to the predominance of frozen oil drops. Upon heating to 40 °C, a fraction of these drops coalesced with one another. At this temperature, the primary fraction of PO had melted, as illustrated by the significant fading of the background colors in Figure S9e. Subsequently, some drops began to coalesce with the surrounding oily medium and disappeared from the field of view.

**Figure 10:**
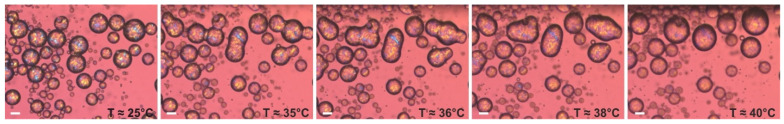
Optical microscopy images, illustrating the process of drop–drop coalescence, observed upon heating of PO drops, dispersed in 1 wt. % HPC-SSL solution. Scale bars = 20 μm.

The second type of model optical experiment was performed using an emulsion sample, prepared with the procedure described in Section 4.2.4, with palm oil dispersed in 1 wt. % HPC-SSL. After preparation, the emulsion sample used for optical observations was diluted with 1 wt. % HPC-SSL and transferred into a rectangular glass capillary. The sample was then stored at 25 °C overnight to facilitate the drop crystallization. Afterwards, this sample was heated at a rate of 5 °C/min under an optical microscope. Figure 10 presents the illustrative results from this experiment. As seen from the images, all drops initially appear as distinct spherical entities, part of which are in contact with one another. Upon heating, the solid bridges connecting neighboring droplets begin to melt, leading to drop–drop coalesce. Eventually, the particles completely melt, forming liquid drops with diameters significantly larger than those of the initial particles.

These results visualize the drop coalescence processes occurring in the samples during storage, ultimately contributing to emulsion destabilization.

## 3. Conclusions

The emulsifying and stabilizing capabilities of six grades of hydroxypropyl cellulose (HPC) polymers, with molecular masses ranging from 40 to 870 kDa, were investigated for the preparation of food emulsion prototypes. The obtained results show that the mean droplet size decreases significantly with an increase in the polymer concentration up to *c* ≈ 2 wt. %, for all studied HPC polymer grades, regardless of polymer molecular mass. At *c* > 2 wt. %, the mean drop size decreases only slightly due to the increased viscosity of the continuous phase. Only about 1% of the polymer molecules adsorb at the oil–water interface during emulsification. Most probably, the adsorbing components have an optimal ratio of hydrophobic to hydrophilic molecular fragments. Further dedicated study would be needed to clarify the actual adsorbing components in the various polymer systems.

The rheological behavior of 25 wt. % (≈ 27 vol. %) palm oil-in-water emulsions at 40 °C is mainly governed by the properties of the polymer solutions. Emulsions prepared with *c* ≥ 3 wt. % HPC-M, HPC-MX or HPC-H appear as highly viscous, shear-thinning liquids or gels. Increasing the oil weight fraction or lowering the temperature promotes drop–drop interactions, forming non-flowing gel-like samples. Stable emulsions form when polymers with *M_w_* ≳ 400 kDa are used at concentrations at least 3.5 times the critical polymer overlap concentration, c*. Under these conditions, small droplets, *d*_32_ ≲ 10 μm, are formed, the viscosity of the continuous phase, *η*_0_ ≥ 1 Pa·s, is sufficient to decelerate creaming, and long-range steric repulsion prevents drop–drop flocculation and coalescence. An exception was the polymer HPC-AW, which could not prevent drop coalescence upon storage.

Lower-molecular-weight HPC polymers, *M_w_* < 90 kDa (grades L and SSL), were unable to stabilize emulsions, even at concentrations up to five times c*, due to insufficient steric repulsion. Films stabilized by M, MX and H polymers remained thick and stable for over 15 min, while those stabilized by L and SSL polymers thinned out quickly and broke. Adding low-molecular-mass surfactants, such as Tween 80^®^, displaced the HPC molecules from the oil–water interface, leading to the formation of thin emulsion films and droplet flocculation and creaming, while preventing drop coalescence and the related bulk oil separation upon emulsion storage.

The physical state of the emulsified drops also affects emulsion stability. Above the oil melting point, the fluid oil drops coalesce, leading to the separation of bulk oil. When the oil is frozen or partially frozen, the drops aggregate without complete merging, thus forming non-flowing gels.

This study provides insights into designing HPC-stabilized food emulsions with tailored rheological characteristics—either freely flowing, highly viscous or gel-like. The findings with palm oil-in-water emulsions are expected to extend to other triglyceride oils rich in saturated fatty acids, such as palm kernel oil, cocoa butter, coconut oil, lard, and others.

## 4. Materials and Methods

### 4.1. Materials

#### 4.1.1. Oils

In the main series of experiments, palm oil (PO, *Elaeis Guineensis Oil*) was used as the dispersed phase; a product of Olineza Premium Ltd., Sofia, Bulgaria was purchased from a local grocery store. Palm oil primarily consists of triglycerides with mixed chain lengths, comprising ca. 48% saturated fatty acid chains (mainly palmitic acid), 42% monounsaturated fatty acids (predominantly oleic acid), and 10% polyunsaturated fatty acid chains, with linoleic acid as the main component [63,64]. This complex triglyceride composition also determines a complex phase behavior, as seen in the differential scanning calorimetry (DSC) thermogram presented in Figure 1b. The DSC experiment was performed with a Discovery DSC 250 apparatus (TA Instruments, New Castle, DE, USA), following the procedure described in Ref. [65]. In the current study, 2 °C/min cooling and heating rates were applied. As seen from the thermogram, the palm oil melting occurs over a broad temperature range, from −10 °C to 43 °C, with several distinct maxima observed at −5.9, 2.7, 12.9 and 33.4 °C. Figure 1b shows that the main fraction of the oil is in a liquid state when the temperature of the sample is maintained above or around 40 °C, whereas around 50% of the oil is in a frozen state at temperatures ≤ 25 °C.

To determine the effect of the solid fraction in PO on the emulsion properties, parts of the emulsions were prepared using sunflower oil (*Helianthus annuus*, see Section 2.3.2), denoted in the text as SFO, with a product of Bunge Romania S.R.L., Buzău, Romania used. Around 90% of the chains in the triglycerides in SFO are unsaturated and the ratio between monounsaturated and polyunsaturated chains is ca. 1:2. This composition determines the significantly lower melting point of ca. −17 °C [66]; therefore, all drops are in a liquid state when stored above this temperature.

#### 4.1.2. Surfactants and Polymers

For the stabilization of the emulsions, we used six hydroxypropyl cellulose (HPC) samples: SSL, L, M, Celny-MX, H (all products of Nippon Soda Co., Ltd., Tokyo, Japan) and AeroWhip^TM^ 625 EZ (product of Ashland Inc., Wilmington, DE, USA, denoted in the text as AW or HPC-AW). The number-average (*M_n_*) and weight-average (*M_w_*) molecular masses determined by the gel permeation chromatography method were *M_n_* ≈ 24 kDa and *M_w_* ≈ 40 kDa for SSL, *M_n_* ≈ 50 kDa and *M_w_* ≈ 84 kDa for L, *M_n_* ≈ 196 kDa and *M_w_* ≈ 394 kDa for M, *M_n_* ≈ 249 kDa and *M_w_* ≈ 455 kDa for MX, *M_n_* ≈ 257 kDa and *M_w_* ≈ 509 kDa for AW, and *M_n_* ≈ 403 kDa and *M_w_* ≈ 870 kDa for H. The polydispersity index varies between 1.67 and 2.16.

In several experiments, we also tested two different hydroxypropylmethyl celluloses (HPMCs), products of Sigma-Aldrich: HPMC with a molecular mass of 10 kDa (product number 423238, Lot #MKCM2740, 1.8–2.0 mol methoxy per mol cellulose, 0.2–0.3 mol propylene oxide per mol cellulose, gel point 58–64°C [67]) and Hypromellose 2910 (product number H3785, Lot #SLBZ2339, viscosity ≈ 4 Pa·s, 2% in H_2_O, *M_n_* ≈ 86 kDa [67]). These HPMCs are denoted in the text as HPMC-A and HPMC-B, respectively. Carboxymethyl cellulose (CMC, the sodium salt of carboxymethyl cellulose, viscosity 1–300 mPa·s at 1% in water), a product from Modernist pantry (Eliot, ME, USA), was also tested.

To compare the emulsifying properties of different polymers to these of the conventional low-molecular-mass surfactants, we used anionic sodium dodecyl sulfate (denoted in the text as SDS) with a purity of 99% (a product of Sigma) and four different polysorbate-type nonionic surfactants (trade name—Tweens, products of Sigma-Aldrich): polyethylene glycol sorbitan monolaurate (Tween 20^®^), polyethylene glycol sorbitan monopalmitate (Tween 40^®^), polyethylene glycol sorbitan monostearate (Tween 60^®^) and polyethylene glycol sorbitan monooleate (Tween 80^®^). They are notated as “T*X*” within the text, where *X* is the number in the Tween name.

### 4.2. Methods

#### 4.2.1. Gel Permeation Chromatography (GPC)

The molecular weights of the studied HPC polymers were determined via a combined GPC/multi-angle laser light scattering (MALLS) system. A Waters Alliance HPLC (Waters Corp., Milford, MA, USA) chromatographic system with an e2695 separation module and two detectors, an Optilab T-rEX DRI (refractive index detector) and a DAWN HELEOS II MALLS (Wyatt Technology, Santa Barbara, CA, USA), were used. The emitting laser operated at a wavelength of 658 nm. The chromatographic separation was achieved by two Ultrahydrogel columns (Waters Corp., Milford, MA, USA) with pore sizes of 12 and 100 nm. Analyses were performed in a 0.05 wt. % NaN_3_ aqueous solution at 30 °C and a flow rate of 0.8 mL/min. The molecular masses were calculated by ASTRA 6 software (Wyatt Technology, Santa Barbara, CA, USA). A refractive index increment (dn/dc) of 0.14 mL/g was used for the calculations [68].

#### 4.2.2. Polymer Precipitation Temperature (PT) Determination

The precipitation temperature of HPC polymers used for emulsion preparation was determined from rheological experiments with the aqueous HPC solutions [28,29]. The experiments were performed at Discovery HR-3 and HR-20 rheometers (TA Instruments, New Castle, DE, USA) using cone and plate geometry (40 mm cone diameter, 1° cone angle). We measured the viscosity of the solutions at a fixed shear rate of 10 s^−1^, as a function of temperature, using a temperature ramp protocol at 1 °C/min heating rate from 25 °C to 60 °C. Illustrative experimental results from these experiments are presented in Figure 1a and Figure S1 in the Supplementary Materials. The *PT* for HPC-L, M, MX, H and AW was determined from such rheology measurements as the intercept point between the linear fits of the viscosity measured at low temperatures and high temperatures; see the dashed lines in Figure S1 in the Supplementary Materials. The *PT* of HPC-SSL was determined by visual observations of the aqueous solution upon slow heating. At least three independent experiments were performed for each polymer.

#### 4.2.3. Rheological Measurements

All rheological measurements for solutions having an apparent viscosity higher than 10 mPa·s at a shear rate of 1 s^−1^ were performed with cone and plate geometry (40 mm cone diameter and 1° cone angle) using Discovery HR-3 and HR-20 rheometers (TA Instruments, New Castle, DE, USA). The viscosity of the HPC polymer solutions and of the prepared emulsions was measured in flow ramp experiments at a fixed temperature of 40 °C, varying the shear rate, $\dot{\gamma }$, between 1 and 7000 s^−1^. For all data reported in the manuscript, we present the average result along with the error bars, representing the calculated standard deviation. Note that most of the results show very good reproducibility and the error bars are within the size of the symbols.

The storage (*G*′) and loss (*G*″) moduli of the HPC aqueous solutions and emulsions were also determined using oscillatory amplitude sweep tests at a fixed frequency of 1 Hz, varying the applied strain between 0.1 and 100%. These measurements were performed at fixed temperatures of 25 °C or 40 °C. Parallel plates geometry (40 mm diameter of the upper plate) was used in part of the experiments with sandpaper (grade P100) attached to both upper and lower plates to prevent a possible wall-slip. The gap was set to 500 μm.

The viscosities of more diluted solutions, which were not appropriate for rheometry measurements, were determined using the bulk viscometer Brookfield DV-II+Pro (Brookfield Engineering Laboratories, Inc, Middleboro, MA, USA). Cylindrical spindels with diameters of 19 and 25 mm were used. The spindels were placed in a thermostated cup with an inner diameter of 28 mm. The measurements were performed at 40 °C.

#### 4.2.4. Aqueous Solution Preparation

The polymer solutions were prepared by dissolving the polymer powders in deionized water, purified with an Elix^®^ Essential 3 water purification system (Merck KGaA, Darmstadt, Germany). To reduce the homogenization time required for complete polymer dissolution, the polymer powders were first suspended in preheated water with a temperature of ca. 60 °C under continuous stirring with a magnetic stirrer. Once a homogeneous suspension was obtained, the glass beaker was transferred to a second water bath with the temperature maintained at ca. 25 °C. Stirring continued until completely transparent, isotropic solutions were obtained.

In certain model experiments, low-molecular-mass surfactants were combined with the polymers. In these cases, the surfactant was first dissolved in water, and the obtained solution was heated before the polymer was added.

All concentrations given throughout the manuscript refer to the surfactant/polymer concentrations in the aqueous phase (not to the entire emulsion).

#### 4.2.5. Emulsion Preparation

The oil-in-water emulsions investigated in this study were prepared using an Ultra-Turrax T25 rotor-stator homogenizer (IKA, Staufen, Germany) operating at a rotation speed of 13 500 rpm for 5 min. Prior to emulsification, both the aqueous and oily phases were equilibrated at 40 °C to ensure that the PO was in a liquid state. This temperature was maintained during the emulsification process. The aqueous phase was placed in a beaker, and the homogenizer was switched on. The oil was then gradually added to the aqueous phase over a ca. 30 s period under continuous stirring. The same protocol was applied for emulsions prepared with sunflower oil and/or low-molecular-weight surfactants.

Most emulsions were formulated with 25 wt. % PO as the dispersed phase. To assess the influence of oil concentration, a limited number of samples with higher oil content were also prepared (see Section 2.2.2). Furthermore, selected formulations were investigated where SFO was used as the dispersed phase instead of PO (see Section 2.3.2). No additional components were included in the formulations (except for several selected samples in which low-molecular-weight surfactants were also used; see Section 2.3.2 and Section 2.4.2). A matrix showing an overview of the studied formulations is presented in Table S1 in the Supplementary Materials.

#### 4.2.6. Drop Size Determination

The drop size distribution of the obtained emulsions was determined using optical microscopy images. A sample of the prepared emulsion was placed in a glass capillary with a rectangular cross-section (100 μm height) and observed with an AxioImager.M2m microscope (Zeiss, Oberkochen, Baden-Württemberg, Germany) at 40 °C in transmitted white light at 50× magnification. The sizes of at least 1000 individual droplets were measured, and the mean volume–surface diameter, *d*_32_, was calculated, $d_{32}=\sum_{i}d_{i}^{3}N_{i}/\sum_{i}d_{i}^{2}N_{i}$, where *N_i_* is the number of drops with the diameter *d_i_*. For all reported results, the average drop size was determined from the measurements of at least three independently prepared emulsion samples. The error bars in the manuscript represent the standard deviation calculated across these independent measurements.

#### 4.2.7. Stability Tests

The stability of the emulsions was assessed through prolonged shelf-storage under gravitational conditions and via centrifugation at 25 °C and 40 °C.

For storage stability, emulsion samples were placed in glass cylinders (inner diameter ≈ 1.5 cm, sample height ≥ 14 cm) and stored at the two studied temperatures for periods ranging from 1 to 6 months. Pictures of the cylinders were taken at regular intervals to monitor changes, and the emulsion behavior was evaluated and classified as described in Section 2.3.1 above. At least three independently prepared samples were analyzed for each composition to ensure reproducibility.

The stability of the most stable samples under gravitational conditions was further investigated using accelerated stability tests via centrifugation. As previously demonstrated [56,57,58,59], centrifugation serves as a reliable method for predicting long-term stability. In this study, a Sigma 3–16 PK centrifuge (Sigma Laborzentrifugen GmbH, Osterode am Harz, Germany) equipped with a rotor № 11133 was employed at a centrifugal acceleration of 1000 g. Freshly prepared emulsion samples (40–45 g) were centrifuged for a pre-determined period of time (1 to 4 h) at either 25 °C or 40 °C. Pictures of the samples were taken hourly to visually assess the appearance of the samples and detect any signs of instability.

#### 4.2.8. Interfacial Tension Measurements

The interfacial properties of the studied solutions were evaluated by interfacial tension measurements at the oil (PO or SFO)–HPC aqueous solution interface using the pendant drop method [69,70]. These experiments were performed with a DSA 30 instrument equipped with a TC40 temperature-controlled cell (both products of Krüss Scientific, Hamburg, Germany). All measurements were carried out at 40 °C, with an oily droplet immersed in a 1 wt. % aqueous HPC solution. For comparison, sodium dodecyl sulfate was tested at a concentration of 3 wt. %.

#### 4.2.9. Model Experiments

- *Thin liquid films*

The properties of the emulsion films formed between two droplets in close contact were investigated using model experiments conducted in a Scheludko–Exerowa cell [60]. Briefly, the cell consisted of a short vertical capillary, where the studied film was formed and a side capillary where the liquid from which the film was formed was filled. Using a pressure control system, connected to the side capillary, one can control the amount of liquid in the film holder, thus forming a foam or emulsion film with a controlled radius. A detailed description of the film formation can be found in Refs. [61,62]. Thin aqueous films were studied in both air (foam films) and in an oily phase (emulsion films) at 40 °C for 15 min in reflected light using a Leica DM RXE optical microscope equipped (Leica Camera AG, Wetzlar, Germany) with a long-distance Nplan ×20/0.4 objective. To prevent condensation and water evaporation during foam film observations, the experimental cell was sealed with a heated glass cover. The capillary cell had a diameter of ≈0.25 mm.

- *Microscopy observations*

To visualize the coalescence process occurring in the samples during storage, we performed model optical microscopy experiments using emulsion samples placed in glass capillaries. The capillaries were enclosed in a custom-made aluminum cell connected to a water circulator (Julabo CF30, Julabo GmbH, Seelbach, Germany), ensuring a precise temperature control. The detailed description of this experimental set-up can be found in our previous studies [71,72].

Optical microscopy observations were performed using an AxioImager.M2m microscope (Zeiss, Oberkochen, Baden-Württemberg, Germany) in transmitted, cross-polarized white light. A lambda (compensator) plate was positioned after the sample and before the analyzer, oriented at a 45° angle relative to both the polarizer and analyzer. Under these conditions, fluid objects appear with magenta color, whereas birefringent objects display brighter, distinct colors [72]. The exact temperature protocols applied during the microscopy experiments are described in Section 2.4.3.

## Figures and Tables

**Table 1:** HPC polymer characteristics. Weight average molecular mass (*M_w_*), precipitation temperature (*PT*) and critical overlap concentration (c*).

HPC Polymer	*M_w_*, kDa	*PT*, °C	c*, wt. %
SSL	40	51.0 ± 0.8	6.5
L	84	49.6 ± 0.7	2.9
M	394	45.4 ± 0.7	0.9
MX	455	48.3 ± 0.8	0.6
AW	509	42.6 ± 0.4	0.6
H	870	44.3 ± 0.3	0.4

**Table 2:** Stability of 25 wt. % PO emulsions, observed after 1 month of storage at 40 °C. The concentrations given in the second-last column are expressed in wt. % with respect to the aqueous phase. For example, “5 ≤ L ≤ 10” notation denotes that the respective behavior is observed for the studied emulsions with HPC-L in the concentration range between 5 and 10 wt. % HPC-L.

Creaming	Partial Coalescence	Coalescence	Systems in This Group, *c* in wt. %	Example in Figure 5
No	No	No	M ≥ 3; MX ≥ 2; H ≥ 2	a
Yes	No	No	5 ≤ L ≤ 10; 1 ≤ SDS ≤ 3	b
Yes/No		Yes/No	15 L; 2 M; 6 CMC	c
No	No	Yes	20 SSL	d
Yes	Yes	No	0.5 H; 1 H; 1 ≤ AW ≤ 3; 10 ≤ SSL ≤ 15; 3 HPMC-B; 1 HPMC-A; 3 ≤ T80 ≤ 5	e
Yes		Yes	2 SSL; L ≤ 2; 1 M; 1 MX	f

## Data Availability

The original contributions presented in this study are included in the article/Supplementary Materials. Further inquiries can be directed to the corresponding author.

## Supplementary Materials

The following supporting information can be downloaded at: https://www.mdpi.com/article/10.3390/gels11020113/s1, Figure S1: Examples of rheological results used for determination of HPC precipitation temperatures; Figure S2: Storage (*G*′, empty symbols) and loss (*G*″, full symbols) moduli measured for HPC solutions as a function of the polymer concentration; Figure S3: (a) Adsorption calculations. (b) Rheological measurements of HPC-M and HPC-MX solutions and serums separated after centrifugation; Figure S4: Average drop diameter *d*_32_ in 25 wt. % PO emulsions stabilized by HPC polymers, as a function of the aqueous solution viscosity; Figure S5: (a) Apparent viscosities of emulsions prepared with 25 wt. % PO and various polymers as a function of the applied shear rate. (b) Emulsion viscosity divided by solution viscosity (viscosity ratio) measured at 10 s^−1^; Figure S6: Viscosity as a function of shear rate measured for the various aqueous solutions; Figure S7: Results from centrifugation experiments performed at 25 °C with 25 wt. % PO emulsions; Figure S8: Interfacial tension measured at 40 °C for 1 wt. % HPC polymers as a function of the time; Figure S9: Optical microscopy pictures taken upon heating of concentrated emulsion. Video S1: Optical microscopy observations performed upon heating of palm oil emulsion stabilized by 2 wt. % HPC-SSL polymer. Drop–drop coalescence and formation of larger-size drops is observed during palm oil melting; see also Figure S9 in the Supplementary Materials. Table S1: Formulation matrix of the studied emulsion samples with HPC polymers.

## Appendix A. Evaluation of Emulsion Stability upon Storage in Gravity Field

The representative results for emulsion stability as a function of HPC-H concentration are shown in Figure A1. These images were taken after 2 days of storage at 40 °C. In the 0.5 wt. % HPC-H-stabilized emulsion, both creaming and coalescence were observed. In contrast, separation of serum was not observed in the 1 wt. % HPC-H-stabilized emulsion, but a thin yellowish layer formed in the upper part of the emulsion due to drop coalescence. Emulsions stabilized with higher concentrations of HPC-H remained stable after emulsification, with no significant changes observed over time.

These results can be explained by considering both the viscosity of the aqueous phase and the average droplet size in the prepared emulsions. The creaming velocity for an emulsion droplet is determined by the balance between the upward gravitational force and the hydrodynamic frictional force which acts in the opposite directions; see, e.g., [54,73]. This velocity depends on drop size, aqueous phase viscosity, the density difference between the drop material and the aqueous phase, and the gravitational constant. Using this simplified approximation, we can estimate that a 20 μm droplet in a 0.5 wt. % HPC-H solution will travel approximately 6 cm/day, whereas in a 1 wt. % HPC-H solution, the velocity reduces to 2 cm/day, and for a 2.5 wt. % HPC-H, it decreases further to around 0.06 cm/day. In this estimate, we used a density difference Δρ = 130 kg/m^3^ and the aqueous phase viscosity measured at 1 s^−1^ shear rate. Therefore, gravitational separation (creaming) is significant in the 0.5 wt. % HPC-H emulsion, but it is nearly completely suppressed in the 2.5 wt. % HPC-H solution.

The stability of the emulsion is also determined by the average and maximal drop sizes. The average drop size, *d*_32_, decreases from ca. 33.2 μm for 0.5 wt. % to 20.1 μm for 1 wt. %, 10.7 μm for 2 wt. % and 7.1 μm for 2.5 wt. % HPC-H emulsions. The maximal drop diameter, represented by the *d*_V95_ value (showing the drop size for which 95% of the oil is dispersed in drops with sizes below *d*_V95_), also decreases significantly with increasing polymer concentration. Specifically, the *d*_V95_ values are ≈70 μm, 54 μm, 26.5 μm and 16.6 μm for 0.5, 1, 2 and 2.5 wt. % HPC-H emulsions, respectively. Therefore, the partial coalescence observed in the 0.5 and 1 wt. % HPC-H emulsions can be attributed to the presence of a small number of relatively large emulsion drops. These larger drops tend to cream rapidly to the top of the container, where they coalesce with one another, eventually forming a bulk oil layer, as illustrated in Figure A1a.

The effect of storage temperature is demonstrated for 1 wt. % HPC-M emulsions stored at 25 and 40 °C in Figure A1b,c. The viscosity of the aqueous phase in this emulsion is comparable to that of the 0.5 wt. % HPC-H, as both concentrations are close to the overlap polymeric concentration, *c**. However, due to the higher concentration of the M polymer, the average drop size is smaller and closely resembles that observed in the 1 wt. % HPC-H emulsion; see the results presented in Figure 2 and Figure 3 above. From the images in Figure A1b,c, it is evident that the 1 wt. % HPC-M emulsions remain stable for 1 day of storage. Over time, the creaming process becomes noticeable, particularly after 7 days of storage. By 14 days, the sample reaches a steady state concerning gravity-driven separation.

The storage temperature determines the coalescence rate in the emulsions. At 40 °C, a small bulk oily layer forms on top of the 1 wt. % HPC-M emulsion after 7 days of storage, accounting for about 10–15% of the PO present in the sample, Figure A1c. After 14 days, the separated oil nearly doubles to around 25% and remains relatively constant afterwards. In contrast, samples stored at 25 °C, as shown in Figure A1b, exhibit no significant changes in appearance after 14 days of storage, when the steady state with respect to the gravitational separation is reached. While the upper part of the emulsion becomes more yellowish after this period, no bulk oil separation occurs as observed in the 40 °C. This difference arises because, at 25 °C, the main part of the PO remains in a frozen state, inhibiting complete drop–drop coalescence. However, since drops contain both liquid and frozen regions, partial coalescence may occur, resulting in a network of gelled, partially coalesced drops (that is why the viscosity of these gel-like samples is significantly higher than that of the fluid samples observed at 40 °C). Therefore, although the emulsion stored at 25 °C appears significantly more stable than its counterpart at 40 °C, heating this sample to 40 °C melts the oil and leads to the formation of a bulk oily layer on top of the emulsion, as demonstrated in Figure A1d, Supplementary Video S1, Figure S9 in the Supplementary Materials, and the discussion in Section 2.4.3 above.
