# Supplementary material for: Hydroxypropyl Cellulose Polymers as Efficient Emulsion Stabilizers: The Effect of Molecular Weight and Overlap Concentration
Source: Gels. 2025 Feb 5;11(2):113. doi: 10.3390/gels11020113 (PMC11854873; doi:10.3390/gels11020113)
Supplement: Supplementary file 1 [file gels-11-00113-s001.zip › Supplementary Materials - HPC polymers.pdf]

Supplementary Materials

Hydroxypropyl cellulose polymers as efficient emulsion stabilizers: the effect of molecular weight and overlap concentration

Diana Cholakova, Krastina Tsvetkova, Viara Yordanova, Kristina Rusanova
Nikolai Denkov, Slavka Tcholakova*

Department of Chemical and Pharmaceutical Engineering
Faculty of Chemistry and Pharmacy, Sofia University,
1 James Bourchier Avenue, 1164 Sofia, Bulgaria

*Corresponding authors:
Prof. Slavka Tcholakova
Department of Chemical and Pharmaceutical Engineering
Sofia University
1 James Bourchier Ave.,
Sofia 1164
Bulgaria
E-mail: sc@lcpe.uni-sofia.bg
Tel: +359 2 8161698

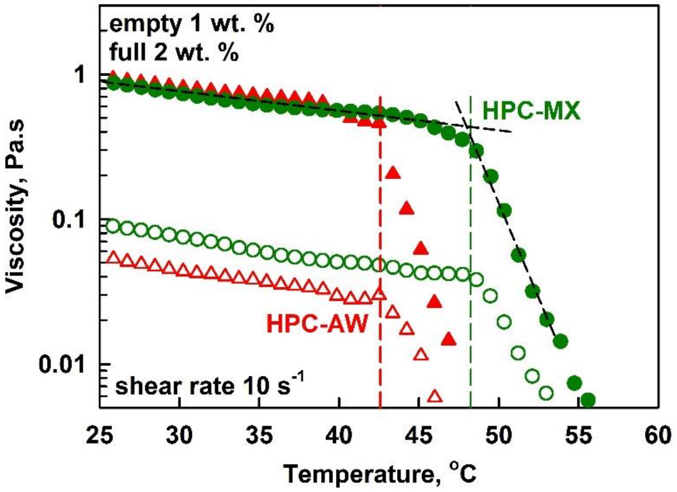

Figure S1. Examples of rheological results used for determination of HPC precipitation temperatures. Results for HPC-MX (green circles) and HPC-AW (red triangles) are shown at concentrations of 1 wt. % (empty symbols) and 2 wt. % (full symbols). The dashed vertical lines show the precipitation temperatures for these samples, where a significant viscosity drop is observed. For AW it is ≈ 42.6°C and for MX ≈ 48.3°C. The viscosities are measured at 10 s^{-1} constant shear rate with 1°C/min heating rate.

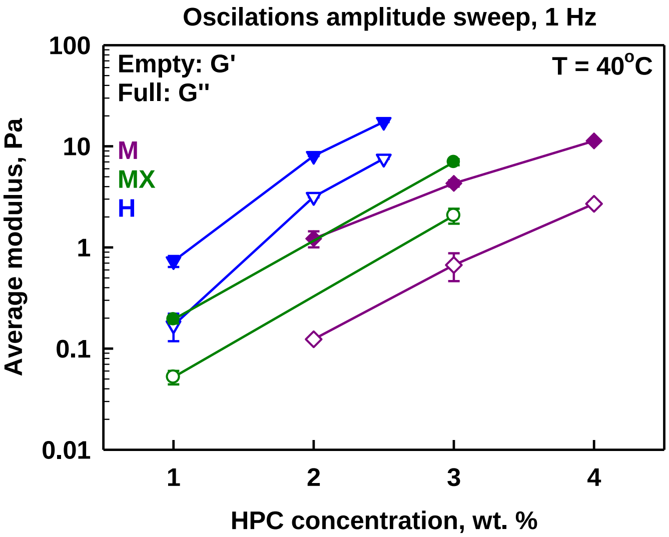

Figure S2. Storage (*G'*, empty symbols) and loss (*G''*, full symbols) moduli measured for HPC solutions as a function of the polymer concentration. All measurements are performed at 40°C and 1 Hz frequency. The average values measured for strains varied between 0.1 and 10 % are shown. As seen from the error bars, representing the standard deviation calculated in the whole strain range, there is no significant dependence from the applied strain.

(a)

Polymer	M_W, kDa	Γ, mg/m²	Γ, μmol/m²	A_M, nm²	A_{MU}, Å²
HPC-SSL	40	281.4	7.04	0.24	0.36
HPC-L	84	122.6	0.88	1.14	0.83
HPC-M	394	123.9	0.18	5.28	0.83
HPC-MX	455	93.5	0.13	8.08	1.09
HPC-H	870	90.6	0.09	15.95	1.13

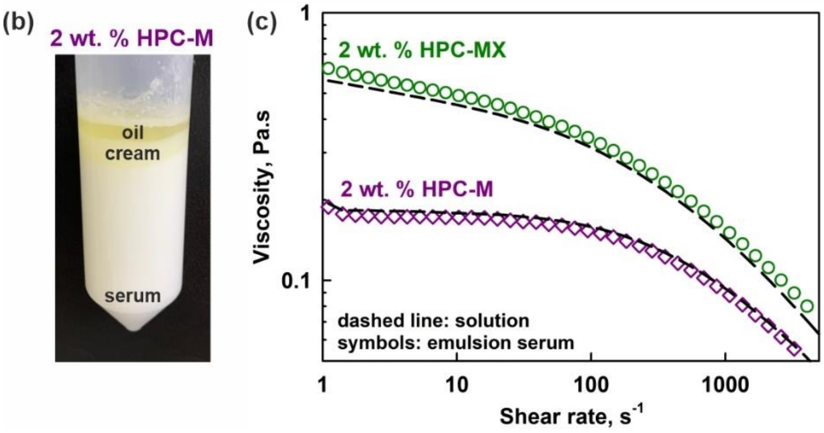

Figure S3. (a) Summary table showing the adsorptions which can be calculated from the $d_{32}(c)$ dependence using eq. 1. However, such high adsorption values have no physical meaning and suggest that the main fraction of the polymer is not adsorbed on the drop surface, but remains in the aqueous solution. Γ denotes the adsorption, A_M is the calculated area per molecule and A_{MU} is the calculated area per monomer unit in the adsorption layer (assuming that 64% of the monomer units in a given molecule are adsorbed on the surface). (b-c) To test this hypothesis, rheological measurements were performed on a serum separated from the respective emulsions after centrifugation, as shown in (b). The comparison between serum viscosity (shown with symbols in c) and the respective aqueous solution viscosity (shown with dashed lines in c) show that these viscosities are practically the same, i.e. the main fraction of the polymer really remains in the aqueous phase of the emulsion and it is not adsorbed on the oil-water interface.

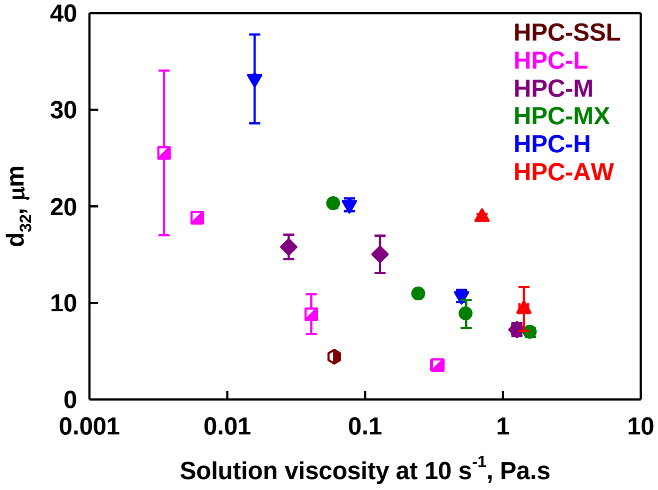

Figure S4. Average drop diameter d_{32} in 25 wt. % PO emulsions stabilized by HPC polymers, as a function of the aqueous solution viscosity. The drop size in emulsions prepared with solutions with higher viscosity is smaller, as compared to the emulsions prepared with solutions of lower viscosity. However, for the same viscosity, for example 60 mPa.s, very different drop sizes are observed with the different polymers, because the viscosity is related to the HPC concentration. These results show that the obtained drop size is primarily determined by the HPC concentration and not by the aqueous phase viscosity.

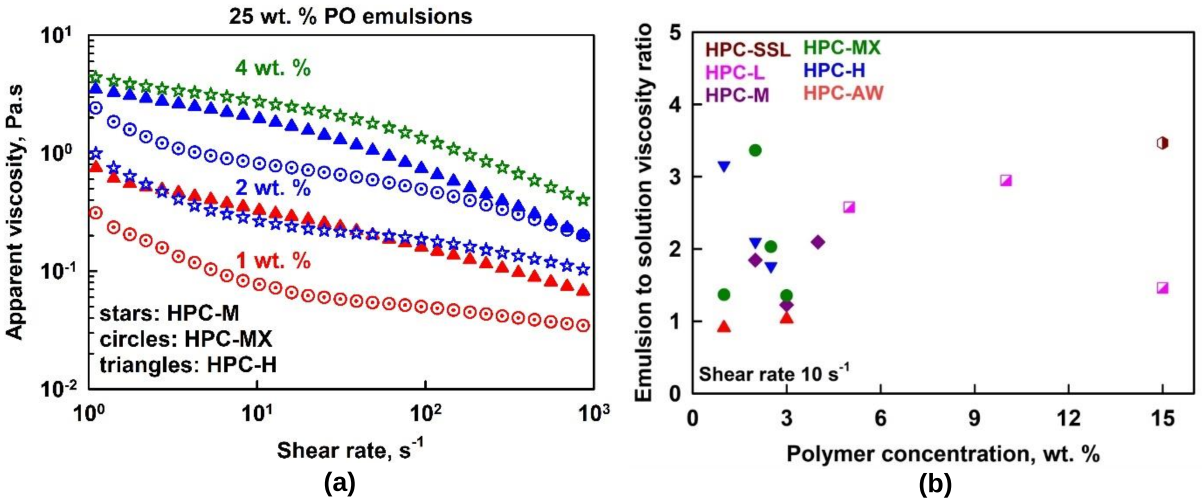

Figure S5. (a) Flow curves for 25 wt. % PO emulsions prepared with different polymers: stars – HPC-M; circles – HPC-MX; triangles – HPC-H. The colors denote the polymer concentration with respect to the aqueous phase: red – 1 wt. %; blue – 2 wt. %; green – 4 wt. %. (b) Emulsion viscosity divided by solution viscosity (viscosity ratio) measured at 10 s^{-1}. There are no clear trends observed in this plot. All rheology measurements are performed at 40°C temperature.

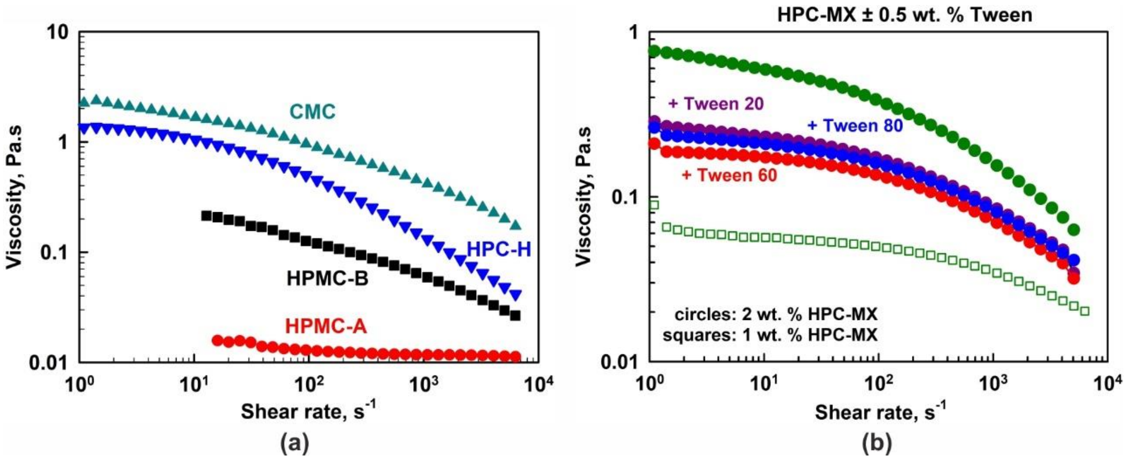

<u>Figure S6.</u> Viscosity as a function of shear rate measured for the aqueous solutions of: (a) 3 wt. % HPMC-A (red circles), 1 wt. % HPMC-B (black squares), 6 wt. % CMC (cyan triangles) and 2 wt. % HPC-H (blue reversed triangles). (b) 1 wt. % HPC-MX (empty green squares); 2 wt. % HPC-MX (full green circles) and 2 wt. % HPC-MX mixed with 0.5 wt. % Tween surfactants: Tween 20 (purple), Tween 60 (red) and Tween 80 (blue).

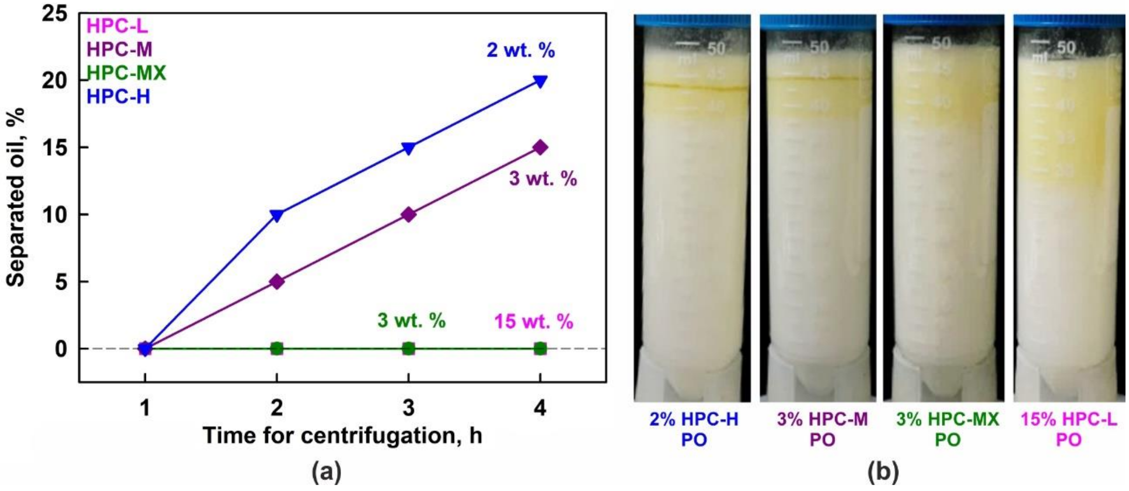

<u>Figure S7.</u> Results from centrifugation experiments performed at 25°C with 25 wt. % PO emulsions. (a) Percent separated oil as a function of the centrifugation time. (b) Appearance of emulsion samples after 3h centrifugation at 25°C.

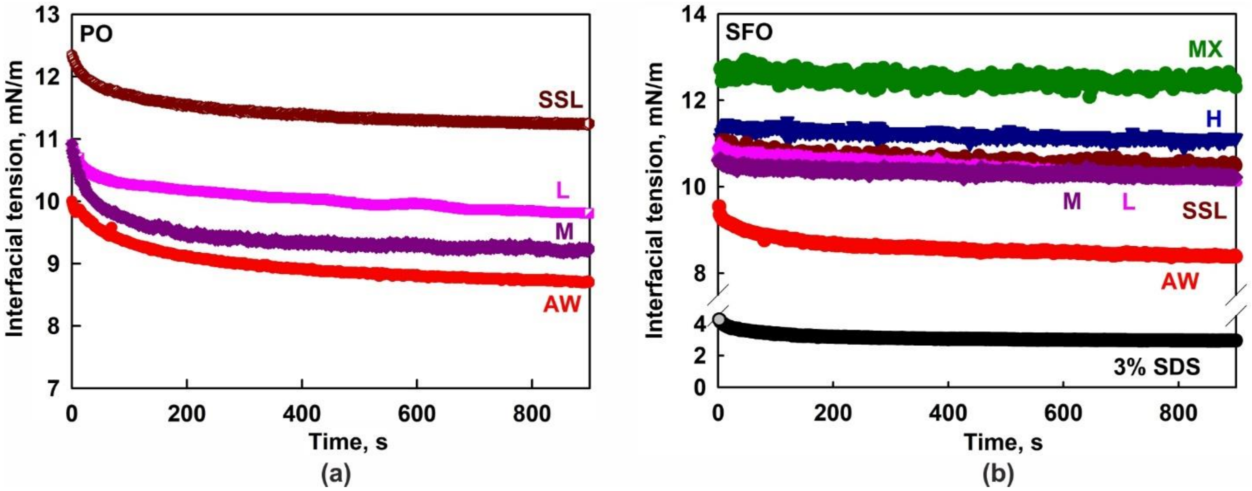

Figure S8. Interfacial tension measured at 40°C for 1 wt. % HPC polymers as a function of the time. The experiments are performed with a pendant drop of (a) palm oil and (b) sunflower oil.

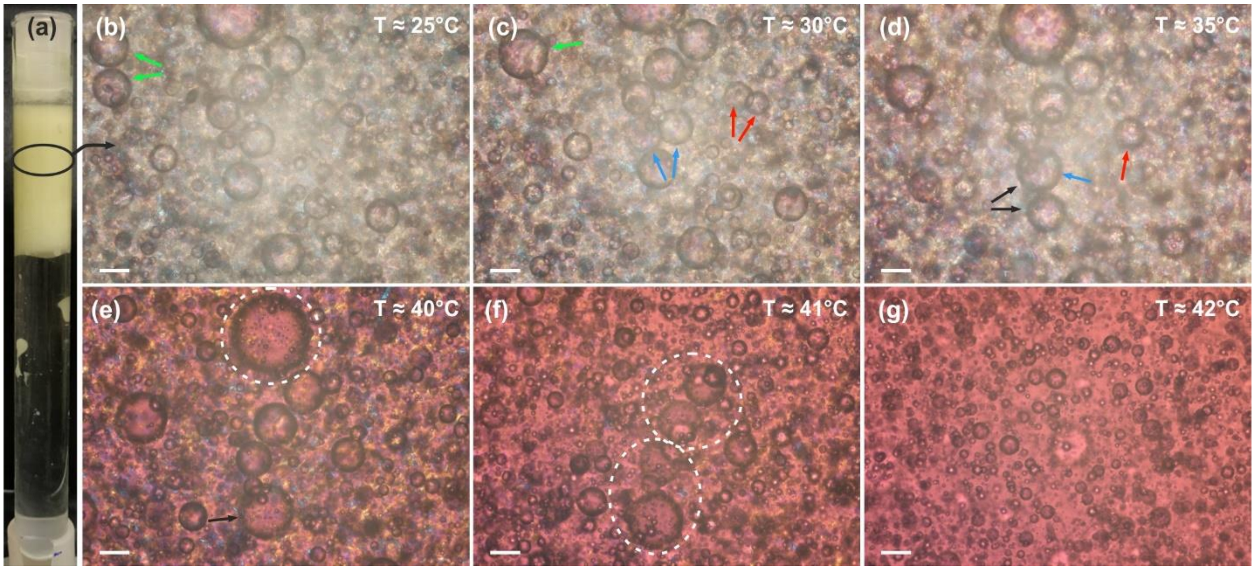

Figure S9. (a) Macroscopic picture of a cylinder filled with PO emulsion stabilized by 2 wt. % HPC-SSL. The emulsion had been stored at 25°C for 5 months. (b-g) Series of optical microscopy images obtained in polarized light upon heating of a sample taken from the emulsion cream shown in (a). In all images, the same region of the sample is observed. (b) Sample observed at 25°C. Note the "greenish" background which is due to the predominant content of frozen oily phase. The two green arrows show two separate drops, which are observed to coalesce upon heating to 30°C, as shown in (c). (c-d) Pictures upon heating show the coalescence of the drops shown with arrows. (e) Picture obtained at 40°C, when the main fraction of PO had melted. Note that many of the big drops present in the sample coalesce at this temperature with the continuous oily phase and disappear from the sample, as shown with dashed circles in (e-g). Scale bars = 50 μm.

Table S1. Formulation matrix of the studied emulsion samples with HPC polymers. In each column for a given oil content, the studied polymers/surfactant concentrations are presented.

**Disperse medium – aqueous solutions of:**	**Dispersed phase**			
	**Palm oil**			**Sunflower oil**
	**25 wt. %**	**50 wt. %**	**60 wt. %**	**25 wt. %**
HPC-SSL	**1 to 20**	**-**	**-**	**-**
HPC-L	**1 to 15**	**-**	**-**	**15**
HPC-M	**1 to 4**	**-**	**-**	**1 to 4**
HPC-MX	**1 to 4**	**-**	**-**	**2; 3**
HPC-MX + Tween ® 20 or 60 or 80	**2 + 0.5**	**-**	**-**	**-**
HPC-AW	**1 to 3**	**-**	**-**	**2; 3**
HPC-H	**0.5 to 3**	**3**	**3**	**1 to 2.5**

** The polymer/surfactant concentrations are presented with respect to the aqueous phase, rather than the entire emulsion.*
